# Vital capacity and inspiratory capacity as additional parameters to evaluate bronchodilator response in asthmatic patients: a cross sectional study

**DOI:** 10.1186/1471-2466-12-49

**Published:** 2012-09-05

**Authors:** Karen S Azevedo, Ronir R Luiz, Patricia RM Rocco, Marcus B Conde

**Affiliations:** 1 Laboratory of Pulmonary Function Tests, Institute of Thoracic Diseases, Federal University of Rio de Janeiro, Rua Professor Rodolpho Paulo Rocco, 255/3º andar/03 F91, Cidade Universitária, CEP 21941-913, Rio de Janeiro, Brazil; 2 Institute of Public Health Studies, Federal University of Rio de Janeiro, Praça Jorge Machado Moréia 100, Cidade Universitária, CEP 21941-598, Rio de Janeiro, Brazil; 3 Laboratory of Pulmonary Investigation, Carlos Chagas Filho Biophysics Institute, Federal University of Rio de Janeiro, Cidade Universitária - CCS, CEP 21941-902, Rio de Janeiro, Brazil; 4 Institute of Thoracic Diseases, Federal University of Rio de Janeiro, Rua Professor Rodolpho Paulo Rocco, 255/6º andar, Cidade Universitária, CEP 21941–913, Rio de Janeiro, Brazil

**Keywords:** Vital capacity, Air trapping, Hyperinflation, Asthma, Bronchodilator response

## Abstract

**Background:**

Bronchodilator response in patients with asthma is evaluated based on post-bronchodilator increase in forced expiratory volume in one second (FEV_1_) and forced vital capacity (FVC). However, the need for additional parameters, mainly among patients with severe asthma, has already been demonstrated.

**Methods:**

The aim of this study was to evaluate the usefulness of vital capacity (VC) and inspiratory capacity (IC) to evaluate bronchodilator response in asthma patients with persistent airflow obstruction. The 43 asthma patients enrolled in the study were stratified into moderate or severe airflow obstruction groups based on baseline FEV_1_. All patients performed a 6-minute walk test before and after the bronchodilator (BD). A bipolar visual analogue scale post-BD was performed to assess clinical effect. The correlation between VC and IC and clinical response, determined by visual analogue scale (VAS) and 6-minute walk test (6MWT), was investigated.

**Results:**

Patients in the severe group presented: 1) greater bronchodilator response in VC (48% *vs* 15%, p = 0.02), 2) a significant correlation between VC variation and the reduction in air trapping (Rs = 0.70; p < 0.01), 3) a significant agreement between VC and VAS score (kappa = 0.57; p < 0.01). There was no correlation between IC and the reduction in air trapping or clinical data.

**Conclusions:**

VC may be a useful additional parameter to evaluate bronchodilator response in asthma patients with severe airflow obstruction.

## Background

Asthma is a serious worldwide health issue, but its clinical manifestations can be controlled with appropriate treatment [1]. Currently, a positive bronchodilator response is established based on an increase ≥ 12% and 200 ml in forced vital capacity (FVC) and/or forced expiratory volume in one second (FEV_1_) compared with baseline values following administration of bronchodilators [2]. However, in clinical practice, patients with moderate or severe asthma may refer clinical improvement after bronchodilator use despite a negative bronchodilator test. In patients with chronic obstructive pulmonary disease (COPD), in whom the bronchodilator test is frequently negative, vital capacity (VC) and inspiratory capacity (IC) variation are used as complementary tools in order to evaluate bronchodilator response [3-6]. COPD patients have a persistent airway obstruction that may also be observed in moderate or severe asthma; however, only a few studies so far have evaluated the usefulness of VC and IC to assess bronchodilator response among asthmatic patients [7-13].

The aim of this study was to analyze the usefulness of VC and IC as additional parameters to assess bronchodilator response in asthma patients with moderate or severe airflow obstruction.

## Methods

This study prospectively enrolled patients aged 15 years and older diagnosed with asthma and persistent airway obstruction according to Global Initiative for Asthma (GINA) criteria, and who were clinically stable at the time of enrollment [14]. Subjects were not eligible if they were current or former smokers (≥ 20 packs) or had clinical and/or radiographic evidence of heart failure, uncontrolled systemic arterial hypertension, pregnancy, focal fibrous scarring on chest X-ray with total area ≥ 1 pulmonary lobe, chest wall deformity or articular or neuromuscular disease, morbid obesity or previous lung resection. Also excluded were patients who used short-acting β2-agonist spray, long-acting β2-agonist spray or oral theophylline 8 h, 12 h or 48 h respectively before the pulmonary function tests, and patients experiencing an asthma crisis or exacerbation during the week before the pulmonary function tests. All patients were referred from the Outpatient Asthma Clinic at the Federal University of Rio de Janeiro Institute of Thoracic Diseases (ITD). They were evaluated at the ITD Pulmonary Function between June 19, 2006 and July 30, 2008. Written informed consent was obtained from all participants (or a legally responsible representative when applicable) and the study was approved by the Federal University of Rio de Janeiro Ethics Committee.

All patients answered a standardized interview and underwent physical examination as well as lateral decubitus and posteroanterior chest x-ray before the bronchodilator test [14]. Pulmonar function tests (PFTs) and a 6-minute walk test (6MWT) were performed before and 15 minutes after the bronchodilator test. Only one 6MWT was done before and after the bronchodilator. A bipolar visual analogue scale (VAS) was used to assess the perceived effect of the bronchodilator test on shortness of breath. The bronchodilator test was performed with salbutamol/400 μg spray under physician supervision (KRSA). PFTs included flow-volume and volume-time curves, inspiratory slow vital capacity maneuver and determination of static lung volumes using a Jaeger spirometer (model MasterScreen-PTF, Hoechberg, Germany), and were conducted according to American Thoracic Society (ATS)/European Respiratory Society (ERS) guidelines [15,16]. Static lung volumes were calculated using the closed-circuit helium dilution method. For He-derived TLC, the end-of-test criterion (equilibration) was defined as helium concentration change of 0.02% or less during 30s rebreathing. The predicted normal values for spirometry and lung volumes were those of Knudson et al. [17]. Polgar/Promadhat and Goldman/Becklake [18,19] respectively. The 6MWT was performed following ATS guidelines, with dyspnea score based on a Borg scale [20].

For the VAS, patients were asked to indicate on a 100 mm horizontal line, labeled “very much worse” on the left end (−100), “very much better” on the right end (+100) and “no change” (zero) in the middle, after the bronchodilator test [21]. Bronchodilator response was defined as an increase in FEV_1_ or FCV ≥ 12% of the baseline value plus 200 ml [2], or a decrease in residual volume (RV) ≥ 20% of the predicted value and 300 ml compared with baseline [22]. Clinical bronchodilator response was defined as **6MWD** ≥ 50 meters or at least 30 meters associated with a reduction greater than 2 points in the Borg scale score [5,20], or any positive value in VAS. For VC and IC, an increase ≥ 12% and 200 ml compared with baseline indicated a positive bronchodilator test.

Asthma patients were stratified into two groups according to the value of FEV_1_: moderate (60% < FEV1 < 80% of predicted values) and severe airflow obstruction (FEV1 ≤ 60% of the predicted values), according to GINA criteria [14].

### Statistical analysis

The Mann–Whitney test was used for quantitative data, and the chi-square test for qualitative parameters. The correlation between the variation in VC and IC and the variation in residual volume-to-total lung capacity ratio (RV/TLC) was analyzed using Spearman’s correlation test. The agreement between VC and IC responses and clinical response was analyzed using the Kappa coefficient. The classification proposed by Chan and Byrt for the interpretation of Spearman and Kappa values respectively, was adopted [23,24]. The statistical package for the social sciences (SPSS) v. 13.0 was used, and a p < 0.05 was considered significant.

## Results

During the study period, 60 subjects were screened and 43 (72%) were enrolled: 20 had moderate airflow obstruction and 23 had severe airflow obstruction. Demographic, clinical and radiological parameters are presented in Table 1. Spirometry, 6MWT, and VAS were performed in all patients, while static lung volumes were analyzed in 37 patients. Functional data for patients with moderate and severe obstruction before (Pre) and after (Post) the bronchodilator (BD), test are depicted in Table 2. VC, IC, FVC, and FEV_1_ were significantly lower in the group with severe airflow obstruction vs. the group with moderate airflow obstruction. Conversely, pre-BD RV/TLC ratio was increased in the presence of severe airflow obstruction. After BD, a significant increase in VC, IC, FVC and FEV1 was observed in both groups as well as a significant reduction in RV/TLC ratio in severe group. A significant increase in 6MWT distance and a decrease in dyspnea score (Borg scale) were observed after the BD in both groups (Table 3).

**Table 1:** Demographic and clinical characteristics of asthma patients

**Parameters**	**Moderate airflow obstruction (n = 20)**	**Severe airflow obstruction (n = 23)**	***P***
Age (years)	50 (37–61)	56 (45–65)	0.32
Height (cm)	156 (152–163)	156 (153–161)	0.76
Weight (kg)	70.6 (58.7-78.9)	67.6 (54.7-72.2)	0.38
Male/Female	6/14	4/19	0.33
Dyspnea score (Borg scale)	0 (0–3.3)	3 (0–5)	0.12
Treatment (Standard/Others)	13/7	20/3	0.09

Standard treatment: regular inhaled corticosteroids and long-acting β_2_-agonists.

*p* value – Mann Whitney test for quantitative data and Chi-square for qualitative data. Quantitative data are expressed as median (25^th^ percentile-75^th^ percentile).

**Table 2:** Pulmonary function before and after bronchodilator of asthma patients

**Parameters % P or L**	**Moderate airflow obstruction (n = 20)**	**Severe airflow obstruction (n = 23)**	***P *(Mann Whitney)**
VC			
Pre-BD (%P)	93.2 (86.5-101.0)	75.7 (67.3-85.2)	<0.01
Post-BD (%P)	102 (91.4-104.7)	84.9 (76,5-96.7)	0.01
P (Wilcoxon)	0.01	<0.01	
IC			
Pre-BD (L)	1.94 (1.63-2.43)	1.56 (1.24-1.88)	0.01
Post-BD (L)	2.1 (1.73-2.63)	1.76 (1.50-1.94)	0.01
P (Wilcoxon)	0.02	<0.01	
FVC			
Pre-BD (%P)	96.2 (88.1-104.3)	76.6 (65.5-93.9)	<0.01
Post-BD (%P)	105.2 (92.3-109.7)	87.7 (76.8-87.7)	0.02
P (Wilcoxon)	<0.01	<0.01	
FEV_1_			
Pre-BD (%P)	68.2 (65.3-77.5)	48.8 (42.7-55.7)	<0.01
Post-BD (%P)	78.9 (70.7-86.3)	60.8 (51.2-65.2)	<0.01
P (Wilcoxon)	<0.01	<0.01	
TLC			
Pre-BD (%P)	95.8 (91.4-113.5)	92.8 (82.3-105.9)	0.19
Post-BD (%P)	99.2 (89.9-109.4)	95.8 (87.4-104.0)	0.40
P (Wilcoxon)	0.8	0.4	
RV/TLC			
Pre-BD (%P)	108.1 (97.6-123.9)	124.8 (105.0-145.0)	0.06
Post-BD (%P)	101.0 (79.7-122.4)	111.0 (91.9-125.4)	0.18
P (Wilcoxon)	0.1	<0.01	
RV			
Pre-BD (% P)	105.2 (90.0-136.6)	113.0 (103.3-145.4)	0.42
Post-BD (% P)	97.3 (76.3-135.6)	102.6 (87.6-124.3)	0.39
P (Wilcoxon)	0.3	0.05	

BD = bronchodilator, FEV_1_ = forced expiratory volume in one second, FVC = forced vital capacity, IC = inspiratory capacity, L = liters, P = predicted, RV = residual volume, TLC = total lung capacity, VC = vital capacity. Results are expressed as median (25^th^ percentile-75^th^ percentile). *p* value for paired sample – Wilcoxon test.

**Table 3:** 6-minute walk test results before and after bronchodilator of asthma patients

**Parameters**	**Moderate airflow obstruction (n = 20)**	**Severe airflow obstruction (n = 23)**	***P *(Mann Whitney)**
6WTD			
Pre-BD (m)	515 (479–570)	501 (468–537)	0.26
Post-BD (m)	555 (511–588)	519 (480–570)	0.26
P (Wilcoxon)	<0.01	<0.01	
Borg Scale			
Pre-BD	5 (0–6)	5 (3–7)	0.35
Post-BD	3 (0–5)	3 (1–4)	0.86
P (Wilcoxon)	0.03	<0.01	

6WTD = 6 minute walk test distance, m = meters, BD = bronchodilator. Results are expressed as median (25^th^ percentile-75^th^ percentile). *p* value for paired sample – Wilcoxon test.

If considering the ATS/ERS definition of obstructive abnormalities, FEV1/VC < 5th percentile of predicted value, only 2 patients did not fulfill this criterion, although they have FEV1 values below 80%, respectively 79.3 and 69.0%.

VC response was observed in 11 (48%) patients with severe obstruction and three (15%) with moderate obstruction (p = 0.02). No significant changes were observed between the two groups in the percentage of bronchodilator response for the other parameters.

The correlations between the variation in spirometric parameters and the variation in RV/TLC (% predicted values) before and after BD were performed and we observed a significant negative correlation between VC and RV/TLC in the severe airflow obstruction group [Spearmans rank correlation coefficient (Rs) = −0.70, p < 0.01], and between FVC and RV/TLC in patients with moderate obstruction (Rs = −0.55, p = 0.03). Conversely, there was no correlation between IC and RV/TLC. Agreement analyses for functional and clinical parameters after BD use are presented in Table 4. There was a fair agreement between VC (severe group) and FVC (moderate group) response and VAS scores, and a slight agreement between RV response (severe group) and VAS score. There was no agreement between functional parameter responses and improvement in 6MWT. There was a poor correlation (p = 0.35) between variations in VC *versus* variation in VAS in severe obstruction patients, and between IC and VAS (p = 0.28) in moderate group.

**Table 4:** Analysis of agreement between functional and clinical positive responses after bronchodilator use

	**VAS**		**6WT**	
**Parameters**	**Moderate airflow obstruction (n = 20)**	**Severe airflow obstruction (n = 23)**	**Moderate airflow obstruction (n = 20)**	**Severe airflow obstruction (n = 23)**
VC	0.20 (0.14)	0.57 (<0.01)	0.17(0.39)	0.13 (0.54)
IC	0.10 (0.61)	0.22 (0.17)	−0.33(0.14)	−0.15 (0.47)
FVC	0.40 (0.03)	0.34 (0.06)	0.00 (1.0)	0.04 (0.86)
FEV_1_	0.10 (0.65)	0.06 (0.75)	−0.05 (0.80)	−0.05 (0.83)
RV	0.10 (0.61)	0.32 (0.04)	0.20 (0.37)	0.12 (0.55)

6MWT = six-minute walk test, FEV_1_ = forced expiratory volume in one second, FVC = forced vital capacity, IC = inspiratory capacity, RV = residual volume, VAS = visual analogue scale, VC = vital capacity. Results are expressed as Kappa coefficient and *p* value in parenthesis.

Five patients with positive VC and/or IC response presented a negative FVC and/or FEV_1_ response.

## Discussion

In the present study with asthma patients with moderate and severe obstruction, we observed that VC may be a useful complementary parameter to FVC and FEV_1_ to assess bronchodilator response.

Asthma patients were assigned to a moderate or severe airflow obstruction group based on FEV_1_ analysis. In addition, because clinical improvement is the main goal of asthma therapy, VAS (subjective criterion) and 6MWT (objective criterion) were also used to evaluate clinical bronchodilator response in this study [20,21,25].

Paré et al. have described two patterns of response to bronchodilator therapy: predominant increase in expiratory flow rate (flow responders) or FVC (volume responders). Volume responders presented lower expiratory flows and greater degree of air trapping [7]. Newton et al., also reported similar results [22]. More recently, Sorkness et al., studying 287 patients with stable but severe asthma, demonstrated that this group presented prominent air trapping in contrast to individuals with non-severe asthma [12]. Our patients with severe airflow obstruction had both greater VC response in the group and greater air trapping (Table 2).

The 6MWT is considered a good parameter to estimate exercise tolerance in patients with moderate or severe pulmonary impairment, even though it has not been extensively used in asthma patients [20]. In our patients a significant increase in 6WTD as well as a decrease in Borg scale were observed in both groups (Table 3). We made only one 6MWT before and after the BD because the reproducibility of the 6MWD has been considered excellent [20].

The post-BD response of VC was well correlated with the variation in RV/TLC ratio in the severe obstruction group. In this line, O’Donnell et al. studied 84 patients with obstructive disease without response in FEV_1_ and found a good correlation between variation in VC and IC and FRC after use of bronchodilator [6]. Additionally, Newton et al. demonstrated that lung volume improvement was independent of changes in maximal expiratory flows in patients with moderate and severe hyperinflation [22].

Agreement between VAS scores and functional responses was statistically significant in only three parameters, VC and RV in the severe obstruction group and FVC in the moderate obstruction group, without agreement with IC response (Table 4). This is in accordance with previous studies reporting a better correlation between dyspnea and the volume of thoracic gas rather than FEV_1_[9,10,13]. A poor correlation (p = 0.35) was observed between variations in VC *versus* variation in VAS in severe obstruction patients. Probably, the reduction in air trapping is not enough to improve exertional breathlessness without a simultaneous and marked decrease in end-expiratory lung volume. Moreover, the increase in VC and IC may represent unrelated effects of the bronchodilation. VAS has been shown to be sensitive to detect improvement in asthma patients [26].

FEV_1_ is considered a good marker of improvement. However, in patients with severe airflow obstruction, FEV_1_ presents a lack of sensitivity and is a poor predictor of improvement in exercise tolerance [22,27-29]. Moreover, Teeter and colleagues demonstrated that asthma symptoms were poorly correlated with FEV_1_ before and after therapy [30]. Therefore, in addition to FEV_1_, other physiologic parameters, such as lung elastic recoil, should be considered to characterize asthma severity [31].

This study has some limitations that need to be addressed: 1) we were not able to correlate the reduction in air trapping with the increase in IC measured in rest, as previously described during exercises tests [3,9,29] or during methacholine challenge testing [8,11] and 2) static lung volumes were measured using the helium dilution method, even though body plethysmography is considered the most sensitive method [25,32]. However, we opted to use more routine tests because ergospirometry, bronchoprovocation test and plethysmography are not available in most pulmonary function laboratories.

## Conclusions

The present findings suggest that VC may be useful in addition to FVC and FEV_1_ to identify bronchodilator response in asthma patients with severe airflow obstruction.

## Competing interests

The authors declare that they have no competing of interests.

## Authors’ contributions

KA conceived of the study, and participated in data collection and data analysis and wrote the manuscript. RL participated in the design of the study and the statistical analysis. PR and MC participated in its design and coordination, contributed to data interpretation and writing the manuscript. All authors read and approved the final manuscript.

## Pre-publication history

The pre-publication history for this paper can be accessed here:

http://www.biomedcentral.com/1471-2466/12/49/prepub

## References

[B1] Global Initiative for Asthma (GINA) Global Strategy for Asthma Management and Prevention. NHLBI / WHO Workshop Report 2009 National Institutes of Health, National Heart, Lung, and Blood Institute, Bethesda, MD

[B2] Pellegrino R Viegi G Brusasco RO Crapo R Burgos F Casaburi R Coates A Van Der Grinten CPM Gustafsson P Hankinson J Jensen R Johnson DC Macintyre N Mckay R Miller MR Navajas D Pederson OF Wanger J Interpretative strategies for lung function tests Eur Respir J 2005 26 948 68 10.1183/09031936.05.00035205 16264058

[B3] O’Donnell DE Lam M Webb KA Measurement of symptoms, lung hyperinflation, and endurance during exercise in chronic obstructive pulmonary disease Am J Respir Crit Care Med 1998 158 1557 1565 9817708 10.1164/ajrccm.158.5.9804004

[B4] Tantucci C Duguet A Similowski T Zelter M Derenne J-P Milic-Emil J Effect of salbutamol on dynamic hyperinflation in chronic obstructive pulmonary disease patients Eur Respir J 1998 12 799 804 10.1183/09031936.98.12040799 9817148

[B5] Rodrigues JR Pereira CAC Resposta a broncodilatador na espirometria: que parâmetros e valores são clinicamente relevantes em doenças obstrutivas? J Pneumol 2000 27 35 47

[B6] O’Donnell DE Forkert L Webb KA Evaluation of bronchodilator responses in patient with “irreversible” emphysema Eur Respir J 2001 18 914 920 10.1183/09031936.01.00216501 11829096

[B7] Paré PD Lawson LM Brooks LA Patterns of response to inhaled bronchodilators in asthmatics Am Rev Respir Dis 1993 127 680 685 10.1164/arrd.1983.127.6.680 6222679

[B8] Lougheed MD Lam M Forkert L Webb KA O’Donnell DE Breathlessness during acute bronchoconstriction in asthma Am Rev Repir Dis 1993 148 1452 1459 10.1164/ajrccm/148.6_Pt_1.1452 8256884

[B9] Tantucci C Ellaffi M Duguet A Similowski T Derenne J-P Milic-Emili J Dynamic hyperinflation and flow limitation during methacholine-induced bronchoconstriction in asthma Eur Respir J 1999 14 295 301 10.1183/9031936.99.14224599 10515404

[B10] Filippelli M Duranti R Gigliott F Bianchi R Grazzini M Stendardi L Scano G Overall contribution of chest wall hyperinflation to breathlessness in asthma Chest 2003 124 2164 2170 10.1378/chest.124.6.2164 14665496

[B11] Lougheed MD Fisher T O’Donnell DE Dynamic hyperinflation during bronchoconstriction in asthma Chest 2006 130 1072 1081 10.1378/chest.130.4.1072 17035440

[B12] Sorkness RL Bleecker ER Busse WW Calhoun WJ Castro M Chung KF Curran-Everett D Erzurun SC Gaston BM Israel E Jarjour NN Moore WC Peters SP Teague WG Wenzel SE Lung function in adults with stable but severe asthma: air trapping and incomplete reversal of obstruction with bronchodilation J Appl Physiol 2008 104 394 403 17991792 10.1152/japplphysiol.00329.2007

[B13] Sutherland TJT Cowan JO Taylor DR Dynamic hyperinflation with bronchoconstriction Am J Respir Crit Care Med 2008 177 970 975 10.1164/rccm.200711-1738OC 18263799

[B14] Global Initiative for Asthma (GINA) Global Strategy for Asthma Management and Prevention. NHLBI / WHO Workshop Report 2006 National Institutes of Health, National Heart, Lung, and Blood Institute, Bethesda, MD

[B15] Miller MR Hankinson J Brusasco V Burgos F Casaburi R Coates A Crapo R Enright P Van Der Grinten CPM Gustafsson P Jensen R Johnson DC Macintyre N Mckay R Navajas D Pedersen OF Pellegrino R Viegi G Wanger J Standardisation of spirometry Eur Respir J 2005 26 319 338 10.1183/09031936.05.00034805 16055882

[B16] Wanger J Clausen J Coates A Pedersen OF Brusasco V Burgos F Casaburi R Crapo R Enright P Van Der Grinten CPM Gustafsson P Hankinson J Jensen R Johnson D Macintyre N Mckay R Miller MR Navajas D Pellegrino R Viegi G Standardisation of the measurement of lung volumes Eur Respir J 2005 26 511 522 10.1183/09031936.05.00035005 16135736

[B17] Knudson RJ Lebowitz MD Holberg CJ Burrows B Changes in the normal maximal expiratory flow-volume curve with growth and aging Am Rev Respir Dis 1983 127 725 734 6859656 10.1164/arrd.1983.127.6.725

[B18] Polgar G Promadhat V Pulmonar function testing in children. Techniques and standards 1971 WB Saunders Co, Philadelphia

[B19] Goldman HI Becklake MR Respiratory Function Tests. Normal values at median altitudes and the prediction of normal results Am Rev Tuberc Pulm Dis 1959 79 457 467 10.1164/artpd.1959.79.4.457 13650117

[B20] American Thoracic Society ATS Statement: Guidelines for the six-minute walk test Am J Respir Crit Care Med 2002 166 111 117 12091180 10.1164/ajrccm.166.1.at1102

[B21] Noseda A Schmerber J Prigogine T Yernault JC Perceived effect on shortness of breath of an acute inhalation of saline or terbutaline: variability and sensitivity of a visual analogue scale in patients with asthma or COPD Eur Respir J 1992 5 1043 1053 1426213

[B22] Newton MF O’Donnell DE Forkert L Response of lung volumes to inhaled salbutamol in a large population of patients with severe hyperinflation Chest 2002 121 1042 1050 10.1378/chest.121.4.1042 11948031

[B23] Byrt T How good is agreement Epidemiology 1996 7 561 8862998 10.1097/00001648-199609000-00030

[B24] Chan YH Biostatitics 104: Correlation analysis Singapore Med J 2003 44 614 619 14770254

[B25] Barreiro E Gea J Sanjuás C Marcos R Broquetas J Milic-Emili J Dyspnoea at rest and at end of different exercises in patients with near-fatal asthma Eur Respir J 2004 24 219 225 10.1183/09031936.04.00074703 15332388

[B26] Isakovic J Isakovic L Filipovic I The correlation between the Borg score and VAS and lung function parameters in patients with asthma and COPD20th Ann Cong of the European Respiratory Society (ERS), Barcelona, 18–22 sep2010 Eur Respir J 2010 Suppl 54

[B27] Belman MJ Warren C Botnick C Shin JW Inhaled bronchodilators reduce dynamic hyperinflation during exercise in patients with chronic obstructive pulmonary disease Am J Respir Crit Care Med 1996 153 967 975 8630581 10.1164/ajrccm.153.3.8630581

[B28] O’Donnell DE Lam M Webb KA Spirometric correlates of improvement in exercise performance after anticholinergic therapy in chronic obstructive pulmonary disease Am J Respir Crit Care Med 1999 160 542 549 10430726 10.1164/ajrccm.160.2.9901038

[B29] Kosmas EN Milic-Emili J Polychronaki A Dimitroulis I Retsou S Gaga M Koutsoukou A Roussos C Koulouris NG Exercise-induce flow limitation, dynamic hyperinflation and exercise capacity in patients with bronchial asthma Eur Respir J 2004 24 378 384 10.1183/09031936.04.00113003 15358695

[B30] Teeter JG Bleecker ER Relationship between airway obstruction and respiratory symptoms in adult asthmatics Chest 1998 11 272 277 9498938 10.1378/chest.113.2.272

[B31] Wenzel S Severe asthma in adults Am J Respir Crit Care Med 2005 172 149 160 10.1164/rccm.200409-1181PP 15849323

[B32] Quanjer PH Tammeling GJ Cotes JE Pederson OF Peslin R Yernault JC Lung volumes and forced ventilatory flows. Report working party standardization of lung function tests, European Communitu for steel and coal. Official statement of the European Respiratory Society Eur Respir J 1993 16 5 40 8499054

